# Hydrogen: An Endogenous Regulator of Liver Homeostasis

**DOI:** 10.3389/fphar.2020.00877

**Published:** 2020-06-11

**Authors:** Yaxing Zhang, Jingting Xu, Hongzhi Yang

**Affiliations:** ^1^Department of Traditional Chinese Medicine, The Third Affiliated Hospital, Sun Yat-sen University, Guangzhou, China; ^2^Institute of Integrated Traditional Chinese and Western Medicine, Sun Yat-sen University, Guangzhou, China; ^3^Biofeedback Laboratory, Xinhua College of Sun Yat-sen University, Guangzhou, China

**Keywords:** gut microbiota, oxidative stress, inflammation, apoptosis, glucose homeostasis, lipids homeostasis

## Abstract

Basic and clinical studies have shown that hydrogen (H_2_), the lightest gas in the air, has significant biological effects of anti-oxidation, anti-inflammation, and anti-apoptosis. The mammalian cells have no abilities to produce H_2_ due to lack of the expression of hydrogenase. The endogenous H_2_ in human body is mainly produced by anaerobic bacteria, such as *Firmicutes* and *Bacteroides*, in gut and other organs through the reversible oxidation reaction of 2 H^+^ + 2 e^-^ ⇌ H_2_. Supplement of exogenous H_2_ can improve many kinds of liver injuries, modulate glucose and lipids metabolism in animal models or in human beings. Moreover, hepatic glycogen has strong ability to accumulate H_2_, thus, among the organs examined, liver has the highest concentration of H_2_ after supplement of exogenous H_2_ by various strategies *in vivo*. The inadequate production of endogenous H_2_ play essential roles in brain, heart, and liver disorders, while enhanced endogenous H_2_ production may improve hepatitis, hepatic ischemia and reperfusion injury, liver regeneration, and hepatic steatosis. Therefore, the endogenous H_2_ may play essential roles in maintaining liver homeostasis.

## Introduction

Hydrogen (H_2_) is the lightest and diffusible gas molecule. H_2_ is produced as an endproduct of carbohydrate fermentation, and is reoxidized primarily by sulfate-reduction, methanogenesis, and acetogenesis (Wolf et al., 2016). However, due to lack of the functional hydrogenase genes, mammalian cells fail to produce H_2_ themselves; the endogenous H_2_ in mammalian is mainly produced by hydrogenases-containing bacterial species present in human gastrointestinal tract, the respiratory system, mouth and pharynx, vagina, and skin (Zhang et al., 2018b; Tan et al., 2019). Over 200 pathogens and pathobionts, and 70% of microbial species in gastrointestinal tract listed in the Human Microbiome Project encode genes for hydrogenases (Wolf et al., 2016; Benoit et al., 2020). Early in 1975, *Malcolm Dole et al*. firstly reported that exposed hairless albino mice with squamous cell carcinoma to a mixture of 97.5 percent H_2_ and 2.5 percent oxygen (O_2_) at a total pressure of 8 atmospheres for periods up to 2 weeks would cause a marked regression of the skin tumors possibly by neutralizing toxic free radicals (Dole et al., 1975). In 2007, the milestone publication in *Nature Medicine* by *Ikuroh Ohsawa et al*. had confirmed that H_2_ acted as a therapeutic antioxidant by selectively reducing the cytotoxic hydroxyl radicals in PC12 cells, however, H_2_ did not react with other reactive oxygen species (ROS), which possess physiological roles (Ohsawa et al., 2007). Thus, inhalation of H_2_ gas markedly suppressed focal ischemia and reperfusion (I/R)-induced brain injury in rats by buffering the effects of oxidative stress. From then on, researchers have extensively investigated the functions and mechanisms of H_2_, studies indicated that supplement of exogenous H_2_ has the potential abilities to protect against acute or chronic damage of tissues or organs, including brain, heart, blood vessel, lung, stomach, intestine, pancreas, liver, gallbladder, kidney, testis, ovary, breast, eye, ear, bones, skin, *et al*. (Guo et al., 2013; Sun et al., 2013; Zhang et al., 2016a; Zhang et al., 2016b; Ge et al., 2017; Zhang et al., 2017; Frajese et al., 2018; Zhang et al., 2018b; Chen et al., 2019; Tan et al., 2019; Tao et al., 2019). H_2_ dissolved in medium protected PC12 cells against cell death in a dose-dependent manner, as that H_2_ > 25 μM had significant anti-oxidative effect (Ohsawa et al., 2007). The concentrations of H_2_ is about 168 μM in small intestine and 43 μM in the stomach of mice, with similar levels predicted in humans (Benoit et al., 2020), this indicates that the concentration of endogenous H_2_ in human body is significantly higher than that needed for anti-oxidative effect.

## The Exogenous Hydrogen in Liver Diseases

Liver has strong ability to increase and accumulate H_2_ after supplement (Kamimura et al., 2011; Sun et al., 2011; Sobue et al., 2015; Iketani et al., 2017; Yamamoto et al., 2019). Among the organs examined *in vivo*, liver has the highest mean maximum concentration (Cmax, 29.0 ± 2.6 μmol/L) in rats by continuous inhalation of 3% H_2_ (Yamamoto et al., 2019). The concentration of H_2_ in liver peaked approximately 5 min following intraperitoneal injection of 8 ml/kg H_2_ rich saline in mice, and returned to the normal levels 40 min later (Sun et al., 2011). Oral intake of H_2_ rich water rapidly but transiently increased H_2_ concentrations in liver and atrial blood, while H_2_ concentrations in arterial blood and kidney were one-tenth of those in rat liver and atrial blood (Sobue et al., 2015). Mechanistically, hepatic glycogen accumulated H_2_ after oral administration of H_2_ rich water *in vivo*, and the *in vitro* experiment also confirmed that glycogen solution maintained H_2_, explaining why consumption of even a small amount of H_2_ over a short span time efficiently improved various liver diseases in animal models (Kamimura et al., 2011; Ohta, 2014).

The imbalance of redox homeostasis plays an important role in liver homeostasis (Chen et al., 2020). Supplements of exogenous H_2_ by the strategies of drinking H_2_ rich water, intraperitoneal injection of H_2_ rich saline, H_2_ saturated lactate Ringer’s solution infused *via* portal vein, and breathing H_2_ gas *et al*., safeguarded various acute or chronic liver injuries in animal models, for example, hepatic I/R injuries, including hepatic portal vein occluding, partial hepatectomy, and cold I/R in liver transplantation *et al*. (Fukuda et al., 2007; Xiang et al., 2012; Matsuno et al., 2014; Tan et al., 2014; Zhang et al., 2015a; Shimada et al., 2016; Lu et al., 2017; Bai et al., 2018; Ishikawa et al., 2018; Li et al., 2018a; Li et al., 2018b; Zhang et al., 2018a; Ge et al., 2019; Uto et al., 2019; Zhang et al., 2019), bile duct ligation (BDL)- (Liu et al., 2010; Liu et al., 2016), sepsis- (Sun et al., 2011; Iketani et al., 2017; Yan et al., 2019), drugs- (Sun et al., 2011; Koyama et al., 2014; Zhang et al., 2015b; Gao et al., 2016), and carbon dioxide (CO_2_) pneumoperitoneum- (Chen et al., 2018) induced liver injuries, *et al*. by suppressing excessive oxidative stress, inflammation and cell death *et al*. (**Supplementary Table 1**). In addition, H_2_ alleviated chronic intermittent hypoxia (IH)-induced liver injury *via* reducing oxidative stress levels (Yang et al., 2018), and improved chronic IH-induced renal injury through reducing renal iron transporting related proteins expression to alleviate iron overload (Guan et al., 2019). It is known that liver is an essential organ that orchestrates systemic iron balance by producing and secreting hepcidin, which acts as the iron hormone, induces degradation of the iron exporter ferroportin to control iron entry into the bloodstream from dietary sources, iron recycling macrophages, and body stores (Wang and Babitt, 2019). However, it is not known whether H_2_ can modulate liver iron sensing and body iron homeostasis.

The liver is a central hub for lipids metabolism, with uptake, esterification, oxidation and secretion of fatty acids (FAs) all occurring in hepatocytes (Chen, 2015; Gluchowski et al., 2017). Hepatic FAs originate from three sources, plasma non-esterified free FAs (lipolysis in adipocytes and unabsorbed portions of lipoproteins after lipoprotein lipase hydrolysis in peripheral tissues), *de novo* biosynthesis from acetyl CoA derived from different sources, and lipoproteins such as chylomicron remnants (leftover of triacylglycerol (TAGs) from the dietary source) (Chen, 2015). FAs in hepatocytes are esterified with glycerol 3-phosphate to generate TAG or with cholesterol to produce cholesterol esters, which are either stored in hepatic lipid droplets or secreted into the circulation in the forms of very low-density lipoprotein (VLDL) particles (Rui, 2014; Chen, 2015). FAs are also incorporated into phospholipids, which are an essential component of cell membranes, and the surface layer of lipid droplets, VLDL, and bile particles (Rui, 2014). During fasted state, FAs are transported into mitochondria for β-oxidation to generate acetyl CoA, which in mitochondria can be used for the production of ketone bodies (Chen, 2015). H_2_ has the abilities to modulate lipids profiles and functions *in vivo*. H_2_ rich saline decreased plasma total cholesterol (TC) and low-density lipoprotein (LDL) cholesterol levels, and reduced the levels of apolipoprotein (apo) B100 in LDL and apo E in VLDL, improved hyperlipidemia-injured high-density lipoprotein (HDL) functions, including the capacity of enhancing cellular cholesterol efflux and protecting against LDL oxidation, in high-fat diet (HFD)-fed Syrian golden hamsters (Zong et al., 2012). In a before-after self-controlled study, patients with potential metabolic syndrome consuming H_2_ rich water for 10 weeks resulted in decreased serum TC and LDL-cholesterol levels, and apo B100 and apo E levels, improved dyslipidemia-injured HDL functions, including the ability to inhibit LDL oxidation, the ability to suppress TNF-α-induced monocyte adhesion to endothelial cells (ECs) and TNF-α-induced ECs apoptosis, and the ability to stimulate cholesterol efflux from macrophage foam cells (Song et al., 2013). These were further confirmed in patients with hypercholesterolemia in a double-blinded, randomized, and placebo-controlled trial (Song et al., 2015). They found that H_2_ treatment increased plasma HDL3-mediated cholesterol efflux *via* ATP-binding cassette transporter A1 from macrophages *ex vivo*; enhanced HDL antiatherosclerotic functions as that of suppressing LDL oxidation, oxidized-LDL-induced THP-1 monocytes adhesion to ECs, ox-LDL-induced ICAM-1, VCAM-1, and IL-6 expression in ECs, and oxidized-LDL-induced ECs apoptosis; decreased plasma levels of TC and LDL cholesterol, apo B100; and decreased plasma levels of malondialdehyde (MDA), interleukin-6 (IL-6) and TNF-α, increased the activity of superoxide dismutase (SOD) in plasma, and increased the activity of paraoxonase-1 (PON-1), an antioxidant enzyme associated with HDL, in both plasma and HDL3 fractions (Song et al., 2015). Using cigarette smoke exposure mice model, *Qin Shucun et al*. found that H_2_ saturated saline minimized the impaired plasma lipid profiles and HDL functionalities, moreover, improved the impaired process of reverse cholesterol transport (RCT), in which it promoted the efflux of excess cholesterol from peripheral tissues and returned it to the liver for utilization, direct secretion into bile and feces disposal (Zong et al., 2015). Therefore, H_2_ is an essential regulator of lipids profiles, HDL functions and RCT *et al*.

Hepatic glucose production accounts for ~90% of endogenous glucose production, and it is crucial for systemic glucose homeostasis, and the net hepatic glucose production is the summation of glucose fluxes from gluconeogenesis, glycogenolysis, glycogen synthesis, glycolysis, and other pathways (Petersen et al., 2017). H_2_ has been shown to maintain the glucose homeostasis, improve fatty liver diseases in animal models and in human beings. Drinking H_2_ rich water reduced obesity, decreased levels of plasma glucose, insulin, and triglyceride, and improved hepatic oxidative stress in *db/db* mice, and alleviated fatty liver in *db/db* mice and HFD-fed wild-type mice (Kamimura et al., 2011; Jackson et al., 2018). Mechanistically, H_2_ increased O_2_ consumption and CO_2_ production without influencing movement activities, and enhanced the expression of hepatic fibroblast growth factor 21 (FGF21), which functioned to improve carbohydrate and lipid homeostasis (Kamimura et al., 2011; BonDurant and Potthoff, 2018). H_2_ rich saline alleviated streptozotocin (STZ) and HFD-induced nonalcoholic fatty liver disease (NAFLD) in rats, decreased fasting blood glucose and insulin levels, improved insulin sensitivity and glucose tolerance, lowered hepatic TNF-α, IL-1β, 8-hydroxy-2′-deoxyguanosine (8-OHdG), 3−nitrotyrosine levels, and Caspase-3 activity, increased hepatic expression of PPARα, which induced the expression of medium-chain acyl-CoA dehydrogenase and acyl-CoA oxidase 1, the rate-limiting enzymes in mitochondrial and peroxisomal fatty acids β-oxidation, respectively, and PPARγ, which contributed to hepatic steatosis (Zhai et al., 2017; Wang et al., 2020). Intraperitoneal injection of H_2_ gas had the therapeutic effect on methionine-choline-deficient (MCD) diet-induced NAFLD in mice *via* inhibiting hepatic MDA levels and JNK phosphorylation (Zhou et al., 2020). *Daisuke Kawai et al*. revealed that H_2_ rich water improved MCD diet-induced nonalcoholic steatohepatitis (NASH) in mice by decreasing plasma ALT levels, hepatic TNF-α and IL-6, oxidative stress and apoptosis related markers, free fatty acid (FFA) uptake-related gene fatty acid translocase (FAT) (Kawai et al., 2012). H_2_ rich water also reduced tumor numbers and maximum tumor size in STZ-induced NASH-related hepatocarcinogenic mice model (Kawai et al., 2012). However, they found that H_2_ decreased hepatic PPARα and its targeted gene FFA β-oxidation-related gene acyl-CoA oxidase expression in MCD diet-induced NASH mice model (Kawai et al., 2012). Therefore, the regulated effects of H_2_ on PPARα might be dependent on the animal models examined, it is possible that H_2_ regulates hepatic lipid metabolism *via* maintaining the balance of hepatic *de novo* lipogenesis/FFAs uptake and β-oxidation. H_2_ also had the protective effects on chronic-plus-binge ethanol (EtOH) feeding-induced liver injury, possibly by inducing acyl ghrelin to suppress the expression of pro-inflammatory cytokines TNF-α and IL-6 and induce the expression of IL-10 and IL-22, thus activating antioxidant enzymes against oxidative stress (Lin et al., 2017). In human beings, drinking H_2_ rich water improved lipids and glucose metabolism in patients with type 2 diabetes or impaired glucose tolerance (Kajiyama et al., 2008), reduced liver fat accumulation in overweight patients suffering from mild-to-moderate NAFLD (Korovljev et al., 2019), improved liver function and reduced viral load in patients with chronic hepatitis B (Xia et al., 2013), attenuated biological reaction to radiation-induced oxidative stress without compromising anti-tumor effects in patients with liver tumors (Kang et al., 2011), and alleviated liver injury of colorectal cancer patients treated with mFOLFOX6 chemotherapy (Yang et al., 2017). Therefore, exogenous H_2_ has the abilities to regulate hepatic glucose and lipids metabolism, attenuate virus and chemotherapy related liver injuries, and improve I/R or drugs-induced hepatic inflammation and oxidative stresses.

## The Endogenous Hydrogen in Liver Homeostasis

H_2_ has antioxidant activity and, in the healthy colon, physiological concentrations of H_2_ might protect the mucosa against oxidative insults, whereas an impaired H_2_ economy might facilitate inflammation or carcinogenesis (Carbonero et al., 2012). Moreover, the decreased endogenous H_2_ levels might also play essential roles in Parkinson’s disease, cerebral and myocardial I/R injuries, and chronic heart failure pathogenesis, while supplement of exogenous H_2_ may act as a possible therapy for these brain and heart diseases (Fu et al., 2009; Fujita et al., 2009; Shinbo et al., 2013; Yoritaka et al., 2013; Zhai et al., 2013; Hasegawa et al., 2015; Ostojic, 2018; Shibata et al., 2018; Suzuki et al., 2018). In liver, the endogenous H_2_ produced by intestinal flora had the ability to improve Concanavalin A (Con A)-induced hepatitis by decreasing serum TNF-α and IFN-γ, while inhibition of intestinal flora by antibiotics aggravated Con A-induced hepatitis (Kajiya et al., 2009). Feeding diet with 20% high amylose cornstarch enhanced H_2_ generation in intestine, and subsequently alleviated hepatic I/R injury in rats (Tanabe et al., 2012). Lactulose accelerated liver regeneration after hepatectomy in rats by inducing endogenous H_2_ production, which may increase hepatic SOD expression and activity, decrease hepatic MDA, IL-6 and TNF-α levels (Yu et al., 2015). Supplement of exogenous H_2_ by H_2_ rich saline had a similar protective effect as lactulose, in contrast, the antibiotics inhibited the regeneration-promoting effect of lactulose by reducing H_2_ production (Yu et al., 2015). L-arabinose, a naturally occurring plant pentose, elicited gut-derived endogenous H_2_ production and alleviated HFD-induced metabolic syndrome, including reduced body weight gain especially fat weight, alleviated liver steatosis, improved glucose homeostasis, reduced systemic dyslipidemia and inflammation in mice (Zhao et al., 2019). Mechanistically, L-arabinose modulated gene-expressions involved in lipid metabolism and mitochondrial function in key metabolic tissues (Zhao et al., 2019). Therefore, endogenous H_2_ is an essential regulator of liver homeostass, such as improving hepatitis, hepatic I/R injury, liver regeneration, hepatic steatosis as well as glucose and lipids homeostasis.

## Discussion

**Figure 1:**
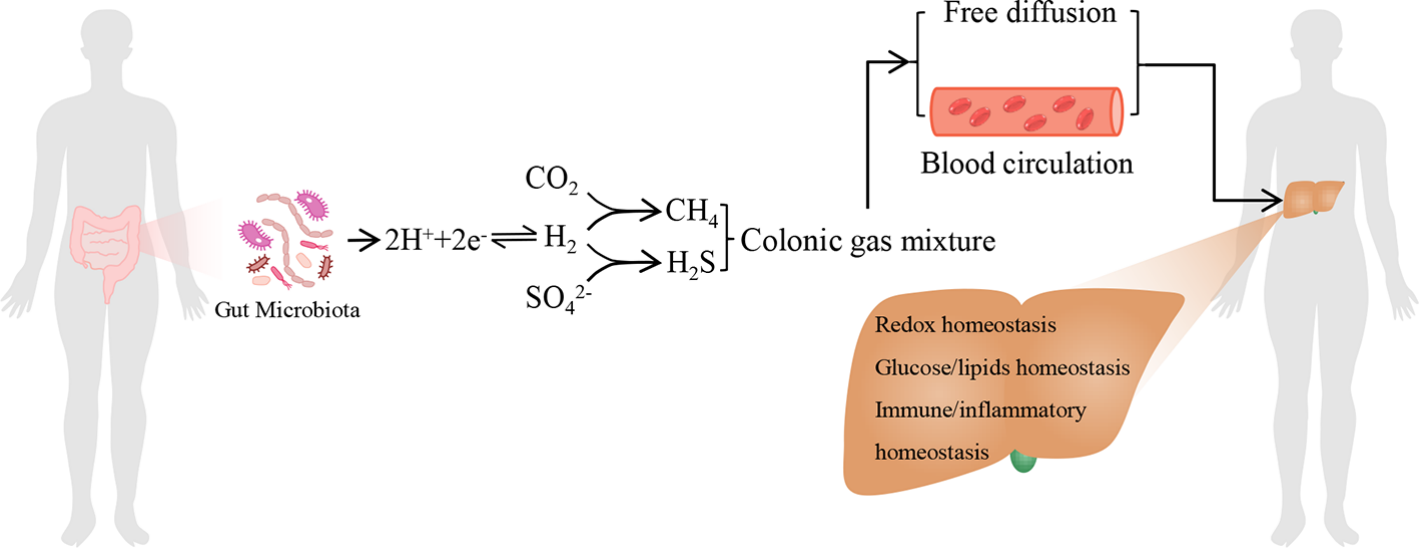
The model of endogenous H_2_ in modulating liver homeostasis. The endogenous H_2_ is primarily produced by hydrogenase-expressing fermentative bacteria in the gastrointestinal tract. Moreover, H_2_S and CH_4_ are by-products of H_2_ metabolism derived from sulphate-reducing bacteria and methanogenic bacteria, respectively. It should be noticed that hepatocyte can also produce H_2_S and CH_4_. The colonic gas mixture, including H_2,_ H_2_S, CH_4_, and other bioactive gas molecules produced by gut microbiota, can arrive at the liver by free diffusion or by blood circulation. These gas molecules may influence hepatic redox homeostasis, glucose and lipids homeostasis, immune and inflammatory homeostasis, respectively, together by another one or more. Therefore, H_2_ may act as a novel bridge between gut and liver, and play a central role among the colonic gas mixture in modulating liver homeostasis.

The total H_2_ levels in mammals are dependent on the balance between H_2_-producing fermentative bacteria, such as colonic *Firmicutes* and *Bacteroidetes et al*., and H_2_ consumers, H_2_ acts as a substrate for acetic acid producing bacteria, methanogenic bacteria, and sulfate reducing bacteria to utilize and support their energy metabolism (Nakamura et al., 2010; Carbonero et al., 2012; Wolf et al., 2016). The H_2_ cycling is central to microbial composition and metabolic homeostasis in the human gastrointestinal tract (Wolf et al., 2016). The gastrointestinal tract-products such as host and/or microbial metabolites (including H_2_) and pathogen-associated molecular patterns translocate to the liver *via* the portal vein or by free diffusion and influence liver functions (Tripathi et al., 2018). In contrast, liver transports bile salts, antimicrobial molecules as well as other liver metabolites to the intestinal lumen through the biliary tract and systemic circulation, some of which maintain microecology balance by controlling unrestricted bacterial overgrowth (Tripathi et al., 2018). Therefore, H_2_ might be as a novel bridge between gut and liver, and play an important role in gut-liver axis (**Figure 1**).

Colonic gases, including H_2_, CO_2_, methane (CH_4_), nitrogen and O_2_ as well as other trace gases including volatile amines, NH_3_, mercaptans, and sulfur-containing gases, such as hydrogen sulphide (H_2_S), which is synthesized by cystathionine γ-lyase (CSE), cystathioine β-synthase (CBS), and 3-mercaptopyruvate sulfurtransferase (3-MST) in concert with cysteine aminotransferase (CAT) in mammalian cells, and is also a by-product of H_2_ metabolism by sulphate-reducing bacteria, are among the most tangible features of digestion, clinically, changes in volume or composition of colonic gases have linked with bowel disorders, including lactose and glucose intolerance, small intestinal bacterial overgrowth (SIBO), irritable bowel syndrome (IBS), inflammatory bowel diseases, constipation, colorectal cancer *et al*., and measurement of H_2_ and CH_4_ by breath can indicate lactose and glucose intolerance, SIBO and IBS (Nakamura et al., 2010; Carbonero et al., 2012; Mani et al., 2014). It should be noticed that both probiotics and harmful bacteria (such as carcinogenic strains of *Helicobacter pylori*) can produce H_2_ (Olson and Maier, 2002; Benoit et al., 2020), therefore, during H_2_ breath test, the relative ratio and balance of these two kinds of bacteria in human body should be taken into consideration for evaluating the long term benefits or harms of H_2_ breath in health.

Similar to the protective effects of endogenous H_2_ on hepatic I/R injury, H_2_S can also act as an endogenous gas molecule that protects against hepatic I/R injury (Hine et al., 2015). Through knockdown or knockout (KO) of H_2_S-generating enzymes in cells or in animals, endogenous H_2_S has been shown to have essential roles in affecting glucose and lipids metabolism, insulin sensitivity, hepatic oxidative stress, hepatic mitochondrial bioenergetics (modulating mitochondrial structure and function, respiratory chain, and cellular bioenergetics), hepatic fibrosis and autophagy *et al*. (Mani et al., 2014; Sun et al., 2015; Ci et al., 2017; Wang et al., 2017; Wenzhong et al., 2017; Untereiner and Wu, 2018; Wu et al., 2019). However, genetically manipulating the H_2_S-producing enzymes in mammalian cells could not exclude the biological effects of endogenous H_2_S produced by sulphate-reducing bacteria in animals. In addition, CH_4_ is another by-product of H_2_ metabolism derived from methanogenic bacteria, and *Mihály Boros* group found that CH_4_ can also be produced by rat liver mitochondria, it has hepatic protective effects by exogenous supplement (Ghyczy et al., 2008; Ye et al., 2015; He et al., 2016; Strifler et al., 2016; Yao et al., 2017; Feng et al., 2019; Li et al., 2019a; Li et al., 2019b), however, the hepatic functions of endogenous CH_4_ produced by methanogenic bacteria or by hepatocytes are not clear. As that H_2_, H_2_S, and CH_4_ are endogenous gas molecules, they may exist as colonic gas mixture and transport into liver by blood circulation or free diffusion, H_2_S and CH_4_ can also be produced in liver. Therefore, the functional crosstalk among H_2_, H_2_S and CH_4_ in liver, and the influences of these gas mixture on hepatic homeostasis are interesting topics for further investigation. It seems that H_2_ might have a central role among these gases in regulating liver homeostasis (**Figure 1**). The temporal and spatial metabolism of microbial H_2_ in human body is relevant to health status, modulating endogenous H_2_ metabolism either by diminished utilization or enhanced production, and the strategies such as developing personalize dietary supplementation and precision medicine based on an individual’s H_2_-producing or consuming microbiome, might provide a novel means of regulating liver homeostasis.

## Author Contributions

Concept and idea: YZ and HY. Preparation of figure and table: YZ, JX. YZ wrote the manuscript. All authors contributed to the article and approved the submitted version.

## Funding

This work was supported by the Natural Science Foundation of Guangdong Province (no. 2018A030313657), the National Natural Science Foundation of China (no. 81900376), the Project funded by China Postdoctoral Science Foundation (no. 2019M653238), and Guangdong famous Traditional Chinese Medicine inheritance studio construction project (no. 20180137).

## Conflict of Interest

The authors declare that the research was conducted in the absence of any commercial or financial relationships that could be construed as a potential conflict of interest.

## References

[B1] Bai G. Li H. Ge Y. Zhang Q. Zhang J. Chen M. (2018). Influence of Hydrogen-rich Saline on Hepatocyte Autophagy During Laparoscopic Liver Ischaemia-reperfusion Combined Resection Injury in Miniature Pigs. J. Vet. Res. 62 (3), 395–403. 10.2478/jvetres-2018-0056 30584622 PMC6295994

[B2] Benoit S. L. Maier R. J. Sawers R. G. Greening C. (2020). Molecular Hydrogen Metabolism: a Widespread Trait of Pathogenic Bacteria and Protists. Microbiol. Mol. Biol. Rev. 84 (1), e00092-19. 10.1128/MMBR.00092-19 31996394 PMC7167206

[B3] BonDurant L. D. Potthoff M. J. (2018). Fibroblast Growth Factor 21: A Versatile Regulator of Metabolic Homeostasis. Annu. Rev. Nutr. 38, 173–196. 10.1146/annurev-nutr-071816-064800 29727594 PMC6964258

[B4] Carbonero F. Benefiel A. C. Gaskins H. R. (2012). Contributions of the microbial hydrogen economy to colonic homeostasis. Nat. Rev. Gastroenterol. Hepatol. 9 (9), 504–518. 10.1038/nrgastro.2012.85 22585131

[B5] Chen M. Jiang L. Li Y. Bai G. Zhao J. Zhang M. (2018). Hydrogen protects against liver injury during CO2 pneumoperitoneum in rats. Oncotarget 9 (2), 2631–2645. 10.18632/oncotarget.23498 29416797 PMC5788665

[B6] Chen J. B. Pan Z. B. Du D. M. Qian W. Ma Y. Y. Mu F. (2019). Hydrogen gas therapy induced shrinkage of metastatic gallbladder cancer: A case report. World J. Clin. cases 7 (15), 2065–2074. 10.12998/wjcc.v7.i15.2065 31423439 PMC6695532

[B7] Chen Z. Tian R. She Z. Cai J. Li H. (2020). Role of oxidative stress in the pathogenesis of nonalcoholic fatty liver disease. Free Radic. Biol. Med. 152, 116–141. 10.1016/j.freeradbiomed.2020.02.025 32156524

[B8] Chen G. (2015). The link between Hepatic Vitamin A Metabolism and Nonalcoholic Fatty Liver Disease. Curr. Drug Targets 16 (12), 1281–1292. 10.2174/1389450116666150325231015 25808650

[B9] Ci L. Yang X. Gu X. Li Q. Guo Y. Zhou Z. (2017). Cystathionine gamma-Lyase Deficiency Exacerbates CCl4-Induced Acute Hepatitis and Fibrosis in the Mouse Liver. Antioxid. Redox Signal 27 (3), 133–149. 10.1089/ars.2016.6773 27848249

[B10] Dole M. Wilson F. R. Fife W. P. (1975). Hyperbaric hydrogen therapy: a possible treatment for cancer. Science 190 (4210), 152–154. 10.1126/science.1166304 1166304

[B11] Feng Y. Cui R. Li Z. Zhang X. Jia Y. Zhang X. (2019). Methane Alleviates Acetaminophen-Induced Liver Injury by Inhibiting Inflammation, Oxidative Stress, Endoplasmic Reticulum Stress, and Apoptosis through the Nrf2/HO-1/NQO1 Signaling Pathway. Oxid. Med. Cell Longev. 2019, 7067619. 10.1155/2019/7067619 31781345 PMC6875424

[B12] Frajese G. V. Benvenuto M. Mattera R. Giampaoli S. Ambrosin E. Bernardini R. (2018). Electrochemically Reduced Water Delays Mammary Tumors Growth in Mice and Inhibits Breast Cancer Cells Survival In Vitro. Evid. Based. Complement. Alternat. Med. 2018, 4753507. 10.1155/2018/4753507 30402124 PMC6196883

[B13] Fu Y. Ito M. Fujita Y. Ito M. Ichihara M. Masuda A. (2009). Molecular hydrogen is protective against 6-hydroxydopamine-induced nigrostriatal degeneration in a rat model of Parkinson’s disease. Neurosci. Lett. 453 (2), 81–85. 10.1016/j.neulet.2009.02.016 19356598

[B14] Fujita K. Seike T. Yutsudo N. Ohno M. Yamada H. Yamaguchi H. (2009). Hydrogen in drinking water reduces dopaminergic neuronal loss in the 1-methyl-4-phenyl-1,2,3,6-tetrahydropyridine mouse model of Parkinson’s disease. PloS One 4 (9), e7247. 10.1371/journal.pone.0007247 19789628 PMC2747267

[B15] Fukuda K. Asoh S. Ishikawa M. Yamamoto Y. Ohsawa I. Ohta S. (2007). Inhalation of hydrogen gas suppresses hepatic injury caused by ischemia/reperfusion through reducing oxidative stress. Biochem. Biophys. Res. Commun. 361 (3), 670–674. 10.1016/j.bbrc.2007.07.088 17673169

[B16] Gao Y. Yang H. Fan Y. Li L. Fang J. Yang W. (2016). Hydrogen-Rich Saline Attenuates Cardiac and Hepatic Injury in Doxorubicin Rat Model by Inhibiting Inflammation and Apoptosis. Mediators Inflammation 2016, 1320365. 10.1155/2016/1320365 PMC5220484 28104928

[B17] Ge L. Yang M. Yang N. N. Yin X. X. Song W. G. (2017). Molecular hydrogen: a preventive and therapeutic medical gas for various diseases. Oncotarget 8 (60), 102653–102673. 10.18632/oncotarget.21130 29254278 PMC5731988

[B18] Ge Y. S. Zhang Q. Z. Li H. Bai G. Jiao Z. H. Wang H. B. (2019). Hydrogen-rich saline protects against hepatic injury induced by ischemia-reperfusion and laparoscopic hepatectomy in swine. Hepatobiliary Pancreat Dis. Int. 18 (1), 48–61. 10.1016/j.hbpd.2018.12.001 30573299

[B19] Ghyczy M. Torday C. Kaszaki J. Szabo A. Czobel M. Boros M. (2008). Hypoxia-induced generation of methane in mitochondria and eukaryotic cells: an alternative approach to methanogenesis. Cell Physiol. Biochem. 21 (1-3), 251–258. 10.1159/000113766 18209491

[B20] Gluchowski N. L. Becuwe M. Walther T. C. Farese R. V. Jr. (2017). Lipid droplets and liver disease: from basic biology to clinical implications. Nat. Rev. Gastroenterol. Hepatol. 14 (6), 343–355. 10.1038/nrgastro.2017.32 28428634 PMC6319657

[B21] Guan P. Sun Z. M. Luo L. F. Zhao Y. S. Yang S. C. Yu F. Y. (2019). Hydrogen Gas Alleviates Chronic Intermittent Hypoxia-Induced Renal Injury through Reducing Iron Overload. Molecules 24 (6), 1184. 10.3390/molecules24061184 PMC6471060 30917568

[B22] Guo J. D. Li L. Shi Y. M. Wang H. D. Hou S. X. (2013). Hydrogen water consumption prevents osteopenia in ovariectomized rats. Br. J. Pharmacol. 168 (6), 1412–1420. 10.1111/bph.12036 23121335 PMC3596646

[B23] Hasegawa S. Goto S. Tsuji H. Okuno T. Asahara T. Nomoto K. (2015). Intestinal Dysbiosis and Lowered Serum Lipopolysaccharide-Binding Protein in Parkinson’s Disease. PloS One 10 (11), e0142164. 10.1371/journal.pone.0142164 26539989 PMC4634857

[B24] He R. Wang L. Zhu J. Fei M. Bao S. Meng Y. (2016). Methane-rich saline protects against concanavalin A-induced autoimmune hepatitis in mice through anti-inflammatory and anti-oxidative pathways. Biochem. Biophys. Res. Commun. 470 (1), 22–28. 10.1016/j.bbrc.2015.12.080 26721437

[B25] Hine C. Harputlugil E. Zhang Y. Ruckenstuhl C. Lee B. C. Brace L. (2015). Endogenous hydrogen sulfide production is essential for dietary restriction benefits. Cell 160 (1-2), 132–144. 10.1016/j.cell.2014.11.048 25542313 PMC4297538

[B26] Iketani M. Ohshiro J. Urushibara T. Takahashi M. Arai T. Kawaguchi H. (2017). Preadministration of Hydrogen-Rich Water Protects Against Lipopolysaccharide-Induced Sepsis and Attenuates Liver Injury. Shock 48 (1), 85–93. 10.1097/SHK.0000000000000810 27918369

[B27] Ishikawa T. Shimada S. Fukai M. Kimura T. Umemoto K. Shibata K. (2018). Post-reperfusion hydrogen gas treatment ameliorates ischemia reperfusion injury in rat livers from donors after cardiac death: a preliminary study. Surg. Today 48 (12), 1081–1088. 10.1007/s00595-018-1693-0 29980846

[B28] Jackson K. Dressler N. Ben-Shushan R. S. Meerson A. LeBaron T. W. Tamir S. (2018). Effects of alkaline-electrolyzed and hydrogen-rich water, in a high-fat-diet nonalcoholic fatty liver disease mouse model. World J. Gastroenterol. 24 (45), 5095–5108. 10.3748/wjg.v24.i45.5095 30568387 PMC6288656

[B29] Kajiya M. Sato K. Silva M. J. Ouhara K. Do P. M. Shanmugam K. T. (2009). Hydrogen from intestinal bacteria is protective for Concanavalin A-induced hepatitis. Biochem. Biophys. Res. Commun. 386 (2), 316–321. 10.1016/j.bbrc.2009.06.024 19523450

[B30] Kajiyama S. Hasegawa G. Asano M. Hosoda H. Fukui M. Nakamura N. (2008). Supplementation of hydrogen-rich water improves lipid and glucose metabolism in patients with type 2 diabetes or impaired glucose tolerance. Nutr. Res. 28 (3), 137–143. 10.1016/j.nutres.2008.01.008 19083400

[B31] Kamimura N. Nishimaki K. Ohsawa I. Ohta S. (2011). Molecular hydrogen improves obesity and diabetes by inducing hepatic FGF21 and stimulating energy metabolism in db/db mice. Obesity (Silver Spring) 19 (7), 1396–1403. 10.1038/oby.2011.6 21293445

[B32] Kang K. M. Kang Y. N. Choi I. B. Gu Y. Kawamura T. Toyoda Y. (2011). Effects of drinking hydrogen-rich water on the quality of life of patients treated with radiotherapy for liver tumors. Med. Gas Res. 1 (1), 11. 10.1186/2045-9912-1-11 22146004 PMC3231938

[B33] Kawai D. Takaki A. Nakatsuka A. Wada J. Tamaki N. Yasunaka T. (2012). Hydrogen-rich water prevents progression of nonalcoholic steatohepatitis and accompanying hepatocarcinogenesis in mice. Hepatology 56 (3), 912–921. 10.1002/hep.25782 22505328

[B34] Korovljev D. Stajer V. Ostojic J. LeBaron T. W. Ostojic S. M. (2019). Hydrogen-rich water reduces liver fat accumulation and improves liver enzyme profiles in patients with non-alcoholic fatty liver disease: a randomized controlled pilot trial. Clin. Res. Hepatol. Gastroenterol. 43 (6), 688–693. 10.1016/j.clinre.2019.03.008 30982748

[B35] Koyama Y. Taura K. Hatano E. Tanabe K. Yamamoto G. Nakamura K. (2014). Effects of oral intake of hydrogen water on liver fibrogenesis in mice. Hepatol. Res. 44 (6), 663–677. 10.1111/hepr.12165 23682614

[B36] Li H. Bai G. Ge Y. Zhang Q. Kong X. Meng W. (2018a). Hydrogen-rich saline protects against small-scale liver ischemia-reperfusion injury by inhibiting endoplasmic reticulum stress. Life Sci. 194, 7–14. 10.1016/j.lfs.2017.12.022 29253502

[B37] Li S. Fujino M. Ichimaru N. Kurokawa R. Hirano S. Mou L. (2018b). Molecular hydrogen protects against ischemia-reperfusion injury in a mouse fatty liver model via regulating HO-1 and Sirt1 expression. Sci. Rep. 8 (1), 14019. 10.1038/s41598-018-32411-4 30232347 PMC6145907

[B38] Li Z. Chen D. Jia Y. Feng Y. Wang C. Tong Y. (2019a). Methane-Rich Saline Counteracts Cholestasis-Induced Liver Damage via Regulating the TLR4/NF-kappaB/NLRP3 Inflammasome Pathway. Oxid. Med. Cell Longev. 2019, 6565283. 10.1155/2019/6565283 31827690 PMC6885841

[B39] Li Z. Jia Y. Feng Y. Cui R. Wang Z. Qu K. (2019b). Methane-Rich Saline Protects Against Sepsis-Induced Liver Damage by Regulating the PPAR-gamma/NF-kappaB Signaling Pathway. Shock 52 (6), e163–e172. 10.1097/SHK.0000000000001310 30601406

[B40] Lin C. P. Chuang W. C. Lu F. J. Chen C. Y. (2017). Anti-oxidant and anti-inflammatory effects of hydrogen-rich water alleviate ethanol-induced fatty liver in mice. World J. Gastroenterol. 23 (27), 4920–4934. 10.3748/wjg.v23.i27.4920 28785146 PMC5526762

[B41] Liu Q. Shen W. F. Sun H. Y. Fan D. F. Nakao A. Cai J. M. (2010). Hydrogen-rich saline protects against liver injury in rats with obstructive jaundice. Liver Int. 30 (7), 958–968. 10.1111/j.1478-3231.2010.02254.x 20492513

[B42] Liu Q. Li B. S. Song Y. J. Hu M. G. Lu J. Y. Gao A. (2016). Hydrogen-rich saline protects against mitochondrial dysfunction and apoptosis in mice with obstructive jaundice. Mol. Med. Rep. 13 (4), 3588–3596. 10.3892/mmr.2016.4954 26936224

[B43] Lu Z. Lin Y. Peng B. Bao Z. Niu K. Gong J. (2017). Hydrogen-Rich Saline Ameliorates Hepatic Ischemia-Reperfusion Injury Through Regulation of Endoplasmic Reticulum Stress and Apoptosis. Dig. Dis. Sci. 62 (12), 3479–3486. 10.1007/s10620-017-4811-8 29086332

[B44] Mani S. Cao W. Wu L. Wang R. (2014). Hydrogen sulfide and the liver. Nitric. Oxide 41, 62–71. 10.1016/j.niox.2014.02.006 24582857

[B45] Matsuno N. Watanabe R. Kimura M. Iwata S. Fujiyama M. Kono S. (2014). Beneficial effects of hydrogen gas on porcine liver reperfusion injury with use of total vascular exclusion and active venous bypass. Transplant. Proc. 46 (4), 1104–1106. 10.1016/j.transproceed.2013.11.134 24815139

[B46] Nakamura N. Lin H. C. McSweeney C. S. Mackie R. I. Gaskins H. R. (2010). Mechanisms of microbial hydrogen disposal in the human colon and implications for health and disease. Annu. Rev. Food Sci. Technol. 1, 363–395. 10.1146/annurev.food.102308.124101 22129341

[B47] Ohsawa I. Ishikawa M. Takahashi K. Watanabe M. Nishimaki K. Yamagata K. (2007). Hydrogen acts as a therapeutic antioxidant by selectively reducing cytotoxic oxygen radicals. Nat. Med. 13 (6), 688–694. 10.1038/nm1577 17486089

[B48] Ohta S. (2014). Molecular hydrogen as a preventive and therapeutic medical gas: initiation, development and potential of hydrogen medicine. Pharmacol. Ther. 144 (1), 1–11. 10.1016/j.pharmthera.2014.04.006 24769081

[B49] Olson J. W. Maier R. J. (2002). Molecular hydrogen as an energy source for Helicobacter pylori. Science 298 (5599), 1788–1790. 10.1126/science.1077123 12459589

[B50] Ostojic S. M. (2018). Inadequate Production of H2 by Gut Microbiota and Parkinson Disease. Trends Endocrinol. Metab. 29 (5), 286–288. 10.1016/j.tem.2018.02.006 29478695

[B51] Petersen M. C. Vatner D. F. Shulman G. I. (2017). Regulation of hepatic glucose metabolism in health and disease. Nat. Rev. Endocrinol. 13 (10), 572–587. 10.1038/nrendo.2017.80 28731034 PMC5777172

[B52] Rui L. (2014). Energy metabolism in the liver. Compr. Physiol. 4 (1), 177–197. 10.1002/cphy.c130024 24692138 PMC4050641

[B53] Shibata A. Sugano Y. Shimouchi A. Yokokawa T. Jinno N. Kanzaki H. (2018). Decrease in exhaled hydrogen as marker of congestive heart failure. Open Heart 5 (2), e000814. 10.1136/openhrt-2018-000814 30245836 PMC6144897

[B54] Shimada S. Wakayama K. Fukai M. Shimamura T. Ishikawa T. Fukumori D. (2016). Hydrogen Gas Ameliorates Hepatic Reperfusion Injury After Prolonged Cold Preservation in Isolated Perfused Rat Liver. Artif. Organs 40 (12), 1128–1136. 10.1111/aor.12710 27140066

[B55] Shinbo T. Kokubo K. Sato Y. Hagiri S. Hataishi R. Hirose M. (2013). Breathing nitric oxide plus hydrogen gas reduces ischemia-reperfusion injury and nitrotyrosine production in murine heart. Am. J. Physiol. Heart Circ. Physiol. 305 (4), H542–H550. 10.1152/ajpheart.00844.2012 23771690

[B56] Sobue S. Yamai K. Ito M. Ohno K. Ito M. Iwamoto T. (2015). Simultaneous oral and inhalational intake of molecular hydrogen additively suppresses signaling pathways in rodents. Mol. Cell Biochem. 403 (1-2), 231–241. 10.1007/s11010-015-2353-y 25707580

[B57] Song G. Li M. Sang H. Zhang L. Li X. Yao S. (2013). Hydrogen-rich water decreases serum LDL-cholesterol levels and improves HDL function in patients with potential metabolic syndrome. J. Lipid Res. 54 (7), 1884–1893. 10.1194/jlr.M036640 23610159 PMC3679390

[B58] Song G. Lin Q. Zhao H. Liu M. Ye F. Sun Y. (2015). Hydrogen Activates ATP-Binding Cassette Transporter A1-Dependent Efflux Ex Vivo and Improves High-Density Lipoprotein Function in Patients With Hypercholesterolemia: A Double-Blinded, Randomized, and Placebo-Controlled Trial. J. Clin. Endocrinol. Metab. 100 (7), 2724–2733. 10.1210/jc.2015-1321 25978109

[B59] Strifler G. Tuboly E. Szel E. Kaszonyi E. Cao C. Kaszaki J. (2016). Inhaled Methane Limits the Mitochondrial Electron Transport Chain Dysfunction during Experimental Liver Ischemia-Reperfusion Injury. PloS One 11 (1), e0146363. 10.1371/journal.pone.0146363 26741361 PMC4720186

[B60] Sun H. Chen L. Zhou W. Hu L. Li L. Tu Q. (2011). The protective role of hydrogen-rich saline in experimental liver injury in mice. J. Hepatol. 54 (3), 471–480. 10.1016/j.jhep.2010.08.011 21145612

[B61] Sun Y. Shuang F. Chen D. M. Zhou R. B. (2013). Treatment of hydrogen molecule abates oxidative stress and alleviates bone loss induced by modeled microgravity in rats. Osteoporos. Int. 24 (3), 969–978. 10.1007/s00198-012-2028-4 22648000

[B62] Sun L. Zhang S. Yu C. Pan Z. Liu Y. Zhao J. (2015). Hydrogen sulfide reduces serum triglyceride by activating liver autophagy via the AMPK-mTOR pathway. Am. J. Physiol. Endocrinol. Metab. 309 (11), E925–E935. 10.1152/ajpendo.00294.2015 26442880

[B63] Suzuki A. Ito M. Hamaguchi T. Mori H. Takeda Y. Baba R. (2018). Quantification of hydrogen production by intestinal bacteria that are specifically dysregulated in Parkinson’s disease. PloS One 13 (12), e0208313. 10.1371/journal.pone.0208313 30586410 PMC6306167

[B64] Tan Y. C. Xie F. Zhang H. L. Zhu Y. L. Chen K. Tan H. M. (2014). Hydrogen-rich saline attenuates postoperative liver failure after major hepatectomy in rats. Clin. Res. Hepatol. Gastroenterol. 38 (3), 337–345. 10.1016/j.clinre.2013.11.007 24502885

[B65] Tan S. Long Z. Hou X. Lin Y. Xu J. You X. (2019). H2 Protects Against Lipopolysaccharide-Induced Cardiac Dysfunction via Blocking TLR4-Mediated Cytokines Expression. Front. Pharmacol. 10, 865. 10.3389/fphar.2019.00865 31440160 PMC6694767

[B66] Tanabe H. Sasaki Y. Yamamoto T. Kiriyama S. Nishimura N. (2012). Suppressive Effect of High Hydrogen Generating High Amylose Cornstarch on Subacute Hepatic Ischemia-reperfusion Injury in Rats. Biosci. Microbiota Food Health 31 (4), 103–108. 10.12938/bmfh.31.103 24936356 PMC4034286

[B67] Tao G. Song G. Qin S. (2019). Molecular hydrogen: current knowledge on mechanism in alleviating free radical damage and diseases. Acta Biochim. Biophys. Sin. (Shanghai) 51 (12), 1189–1197. 10.1093/abbs/gmz121 31738389

[B68] Tripathi A. Debelius J. Brenner D. A. Karin M. Loomba R. Schnabl B. (2018). The gut-liver axis and the intersection with the microbiome. Nat. Rev. Gastroenterol. Hepatol. 15 (7), 397–411. 10.1038/s41575-018-0011-z 29748586 PMC6319369

[B69] Untereiner A. Wu L. (2018). Hydrogen Sulfide and Glucose Homeostasis: A Tale of Sweet and the Stink. Antioxid. Redox Signal 28 (16), 1463–1482. 10.1089/ars.2017.7046 28699407

[B70] Uto K. Sakamoto S. Que W. Shimata K. Hashimoto S. Sakisaka M. (2019). Hydrogen-rich solution attenuates cold ischemia-reperfusion injury in rat liver transplantation. BMC Gastroenterol. 19 (1), 25. 10.1186/s12876-019-0939-7 30736744 PMC6368804

[B71] Wang C. Y. Babitt J. L. (2019). Liver iron sensing and body iron homeostasis. Blood 133 (1), 18–29. 10.1182/blood-2018-06-815894 30401708 PMC6318427

[B72] Wang B. Zeng J. Gu Q. (2017). Exercise restores bioavailability of hydrogen sulfide and promotes autophagy influx in livers of mice fed with high-fat diet. Can. J. Physiol. Pharmacol. 95 (6), 667–674. 10.1139/cjpp-2016-0611 28177674

[B73] Wang Y. Nakajima T. Gonzalez F. J. Tanaka N. (2020). PPARs as Metabolic Regulators in the Liver: Lessons from Liver-Specific PPAR-Null Mice. Int. J. Mol. Sci. 21 (6), 2061. 10.3390/ijms21062061 PMC7139552 32192216

[B74] Wenzhong W. Tong Z. Hongjin L. Ying C. Jun X. (2017). Role of Hydrogen Sulfide on Autophagy in Liver Injuries Induced by Selenium Deficiency in Chickens. Biol. Trace Elem Res. 175 (1), 194–203. 10.1007/s12011-016-0752-x 27216022

[B75] Wolf P. G. Biswas A. Morales S. E. Greening C. Gaskins H. R. (2016). H2 metabolism is widespread and diverse among human colonic microbes. Gut. Microbes 7 (3), 235–245. 10.1080/19490976.2016.1182288 27123663 PMC4939926

[B76] Wu D. D. Wang D. Y. Li H. M. Guo J. C. Duan S. F. Ji X. Y. (2019). Hydrogen Sulfide as a Novel Regulatory Factor in Liver Health and Disease. Oxid. Med. Cell Longev. 2019, 3831713. 10.1155/2019/3831713 30805080 PMC6360590

[B77] Xia C. Liu W. Zeng D. Zhu L. Sun X. Sun X. (2013). Effect of hydrogen-rich water on oxidative stress, liver function, and viral load in patients with chronic hepatitis B. Clin. Transl. Sci. 6 (5), 372–375. 10.1111/cts.12076 24127924 PMC5350887

[B78] Xiang L. Tan J. W. Huang L. J. Jia L. Liu Y. Q. Zhao Y. Q. (2012). Inhalation of hydrogen gas reduces liver injury during major hepatotectomy in swine. World J. Gastroenterol. 18 (37), 5197–5204. 10.3748/wjg.v18.i37.5197 23066313 PMC3468851

[B79] Yamamoto R. Homma K. Suzuki S. Sano M. Sasaki J. (2019). Hydrogen gas distribution in organs after inhalation: Real-time monitoring of tissue hydrogen concentration in rat. Sci. Rep. 9 (1), 1255. 10.1038/s41598-018-38180-4 30718910 PMC6362202

[B80] Yan M. Yu Y. Mao X. Feng J. Wang Y. Chen H. (2019). Hydrogen gas inhalation attenuates sepsis-induced liver injury in a FUNDC1-dependent manner. Int. Immunopharmacol. 71, 61–67. 10.1016/j.intimp.2019.03.021 30877875

[B81] Yang Q. Ji G. Pan R. Zhao Y. Yan P. (2017). Protective effect of hydrogen-rich water on liver function of colorectal cancer patients treated with mFOLFOX6 chemotherapy. Mol. Clin. Oncol. 7 (5), 891–896. 10.3892/mco.2017.1409 29142752 PMC5666661

[B82] Yang S. C. Chen L. L. Fu T. Li W. Y. Ji E. S. (2018). [Improvement of hydrogen on liver oxidative stress injury in chronic intermittent hypoxia rats]. Zhongguo Ying Yong Sheng Li Xue Za Zhi 34 (1), 61–64. 10.12047/j.cjap.5484.2018.016 29926661

[B83] Yao Y. Wang L. Jin P. Li N. Meng Y. Wang C. (2017). Methane alleviates carbon tetrachloride induced liver injury in mice: anti-inflammatory action demonstrated by increased PI3K/Akt/GSK-3beta-mediated IL-10 expression. J. Mol. Histol. 48 (4), 301–310. 10.1007/s10735-017-9728-1 28597201

[B84] Ye Z. Chen O. Zhang R. Nakao A. Fan D. Zhang T. (2015). Methane Attenuates Hepatic Ischemia/Reperfusion Injury in Rats Through Antiapoptotic, Anti-Inflammatory, and Antioxidative Actions. Shock 44 (2), 181–187. 10.1097/SHK.0000000000000385 26009821

[B85] Yoritaka A. Takanashi M. Hirayama M. Nakahara T. Ohta S. Hattori N. (2013). Pilot study of H(2) therapy in Parkinson’s disease: a randomized double-blind placebo-controlled trial. Mov. Disord. 28 (6), 836–839. 10.1002/mds.25375 23400965

[B86] Yu J. Zhang W. Zhang R. Ruan X. Ren P. Lu B. (2015). Lactulose accelerates liver regeneration in rats by inducing hydrogen. J. Surg. Res. 195 (1), 128–135. 10.1016/j.jss.2015.01.034 25700936

[B87] Zhai X. Chen X. Shi J. Shi D. Ye Z. Liu W. (2013). Lactulose ameliorates cerebral ischemia-reperfusion injury in rats by inducing hydrogen by activating Nrf2 expression. Free Radic. Biol. Med. 65, 731–741. 10.1016/j.freeradbiomed.2013.08.004 23954468

[B88] Zhai X. Chen X. Lu J. Zhang Y. Sun X. Huang Q. (2017). Hydrogen-rich saline improves nonalcoholic fatty liver disease by alleviating oxidative stress and activating hepatic PPARalpha and PPARgamma. Mol. Med. Rep. 15 (3), 1305–1312. 10.3892/mmr.2017.6120 28098910

[B89] Zhang C. B. Tang Y. C. Xu X. J. Guo S. X. Wang H. Z. (2015a). Hydrogen gas inhalation protects against liver ischemia/reperfusion injury by activating the NF-kappaB signaling pathway. Exp. Ther. Med. 9 (6), 2114–2120. 10.3892/etm.2015.2385 26136944 PMC4473536

[B90] Zhang J. Y. Song S. D. Pang Q. Zhang R. Y. Wan Y. Yuan D. W. (2015b). Hydrogen-rich water protects against acetaminophen-induced hepatotoxicity in mice. World J. Gastroenterol. 21 (14), 4195–4209. 10.3748/wjg.v21.i14.4195 25892869 PMC4394080

[B91] Zhang Y. Xu J. Long Z. Wang C. Wang L. Sun P. (2016a). Hydrogen (H2) Inhibits Isoproterenol-Induced Cardiac Hypertrophy via Antioxidative Pathways. Front. Pharmacol. 7, 392. 10.3389/fphar.2016.00392 27833552 PMC5081383

[B92] Zhang Y. X. Xu J. T. You X. C. Wang C. Zhou K. W. Li P. (2016b). Inhibitory Effects of Hydrogen on Proliferation and Migration of Vascular Smooth Muscle Cells via Down-Regulation of Mitogen/Activated Protein Kinase and Ezrin-Radixin-Moesin Signaling Pathways. Chin. J. Physiol. 59 (1), 46–55. 10.4077/CJP.2016.BAE365 26875562

[B93] Zhang Y. Long Z. Xu J. Tan S. Zhang N. Li A. (2017). Hydrogen inhibits isoproterenolinduced autophagy in cardiomyocytes in vitro and in vivo. Mol. Med. Rep. 16 (6), 8253–8258. 10.3892/mmr.2017.7601 28944928

[B94] Zhang Q. Ge Y. Li H. Bai G. Jiao Z. Kong X. (2018a). Effect of hydrogen-rich saline on apoptosis induced by hepatic ischemia reperfusion upon laparoscopic hepatectomy in miniature pigs. Res. Vet. Sci. 119, 285–291. 10.1016/j.rvsc.2018.07.005 30077949

[B95] Zhang Y. Tan S. Xu J. Wang T. (2018b). Hydrogen Therapy in Cardiovascular and Metabolic Diseases: from Bench to Bedside. Cell Physiol. Biochem. 47 (1), 1–10. 10.1159/000489737 29763888

[B96] Zhang Q. Piao C. Xu J. Jiao Z. Ge Y. Liu X. (2019). Comparative study on protective effect of hydrogen rich saline and adipose-derived stem cells on hepatic ischemia-reperfusion and hepatectomy injury in swine. BioMed. Pharmacother. 120, 109453. 10.1016/j.biopha.2019.109453 31561069

[B97] Zhao L. Wang Y. Zhang G. Zhang T. Lou J. Liu J. (2019). L-Arabinose Elicits Gut-Derived Hydrogen Production and Ameliorates Metabolic Syndrome in C57BL/6J Mice on High-Fat-Diet. Nutrients 11 (12), 3054–8258. 10.3390/nu11123054 PMC6950088 31847305

[B98] Zhou G. Xu K. Chen Z. Xu J. Dai M. Yang H. (2020). Therapeutic effect of hydrogen on nonalcoholic fatty liver disease in mice and its mechanism. J. Trop. Med. 20 (1), 34–38. 10.3969/j.issn.1672-3619.2020.01.007

[B99] Zong C. Song G. Yao S. Li L. Yu Y. Feng L. (2012). Administration of hydrogen-saturated saline decreases plasma low-density lipoprotein cholesterol levels and improves high-density lipoprotein function in high-fat diet-fed hamsters. Metabolism 61 (6), 794–800. 10.1016/j.metabol.2011.10.014 22153840

[B100] Zong C. Song G. Yao S. Guo S. Yu Y. Yang N. (2015). Cigarette smoke exposure impairs reverse cholesterol transport which can be minimized by treatment of hydrogen-saturated saline. Lipids Health Dis. 14, 159. 10.1186/s12944-015-0160-9 26634341 PMC4668613

