# Supplementary material for: Hydrogen: An Endogenous Regulator of Liver Homeostasis
Source: Front Pharmacol. 2020 Jun 11;11:877. doi: 10.3389/fphar.2020.00877 (PMC7301907; doi:10.3389/fphar.2020.00877)
Supplement: Supplementary file 1 [file Table_1.docx]

Supplementary Table

Table S1. Protective effects of H_2_ in Acute or Chronic Hepatic Injures Animal Models

| Animal Models | Mechanisms | References |
| --- | --- | --- |
| Ischemia/Reperfusion (I/R)-induced liver injury | Hepatic malondialdehyde (MDA), TNF-α, IL-6, IL-1β, CD31, HMGB-1 and Egr-1↓  Serum TNF-α, IL-6 levels↓  Hepatic superoxide dismutase (SOD), Catalase (CAT) activities and glutathione (GSH) content↑  Hepatic A20, Bcl-2, HO-1 and Sirt1↑  Hepatic NF-κB (Model dependent)↑↓  Hepatic p-MKK4 and p-JNK↓  Hepatocyte endoplasmic reticulum (ER) stress, apoptosis and autophagy↓  Liver regeneration markers↑ | (Fukuda et al., 2007; Xiang et al., 2012; Matsuno et al., 2014; Tan et al., 2014; Zhang et al., 2015a; Shimada et al., 2016; Lu et al., 2017; Bai et al., 2018; Ishikawa et al., 2018; Li et al., 2018a; Li et al., 2018b; Zhang et al., 2018; Ge et al., 2019; Uto et al., 2019; Zhang et al., 2019) |
| Bile duct ligation (BDL)-induced liver injury | Hepatic TNF-α, IL-1β, IL-6 and HMGB1↓  Hepatic SOD and CAT activities↑  Hepatic MDA and myeloperoxidase (MPO) contents↓  Hepatic p-ERK1/2↓ | (Liu et al., 2010; Liu et al., 2016) |
| Cecal ligation and puncture (CLP)-induced liver injury | FUNDC1-dependent mitophagy↑ | (Yan et al., 2019) |
| Lipopolysaccharide (LPS)-induced liver injury | Hepatic HO-1↑  Hepatic endothelin-1, 8-hydroxy-2′-deoxyguanosine (8-OHdG) and 4-HNE↓  TUNEL-positive cells↓ | (Iketani et al., 2017) |
| D-galactosamine/LPS-induced liver injury | Macrophages infiltration↓  Serum TNF-α and IL-6 levels↓  Hepatic ROS, Caspase-3 activity, cleaved-PARP, p-JNK↓  Hepatic GSH content↑ | (Sun et al., 2011) |
| Carbon tetrachloride-induced liver injury | Macrophages infiltration↓  Serum TNF-α and IL-6 levels↓  Hepatic ROS, α-SMA, hydroxyproline, and Col1a1 mRNA↓  Hepatic GSH content↑  TUNEL-positive cells↓ | (Sun et al., 2011; Koyama et al., 2014) |
| Diethylnitrosamine-induced liver injury | Serum IL-6 levels↓  Hepatic phosphorylation of c-jun↓  Hepatocyte compensatory proliferation↓ | (Sun et al., 2011) |
| Acetaminophen (APAP)-induced liver injury | Hepatic MDA, MPO, peroxynitrite and 4-HNE↓  Hepatic SOD and glutathione peroxidase (GSH-Px) activities, GSH content↑  Hepatic ER and mitochondria structure injuries↓  Hepatic p-JNK, connexin 32 and CYP2E1↓  Hepatic TNF-α, IL-1β mRNA levels↓  Hepatocyte proliferation↑ | (Zhang et al., 2015b) |
| Doxorubicin (DOX)-induced liver injury | Hepatic ROS, MDA↓  Hepatic TNF-α, IL-1β and IL-6↓  Hepatic Bax, cleaved-Caspase-3/8↓  Hepatic Bcl2↑  TUNEL-positive cells↓ | (Gao et al., 2016) |
| CO_2_ pneumoperitoneum-induced liver injury | Hepatic SOD activity, SOD2 and HO-1 mRNA↑  Hepatic MDA, TNF-α and IL-6↓ | (Chen et al., 2018) |
| Chronic intermittent hypoxia (IH)-induced liver injury | Serum levels of 8-OHdG and IL-6↓  Serum SOD activity↑ | (Yang et al., 2018) |

**References**

Bai, G., Li, H., Ge, Y., Zhang, Q., Zhang, J., Chen, M., et al. (2018). Influence of Hydrogen-rich Saline on Hepatocyte Autophagy During Laparoscopic Liver Ischaemia-reperfusion Combined Resection Injury in Miniature Pigs. *J Vet Res* 62(3)**,** 395-403. doi: 10.2478/jvetres-2018-0056.

Chen, M., Jiang, L., Li, Y., Bai, G., Zhao, J., Zhang, M., et al. (2018). Hydrogen protects against liver injury during CO2 pneumoperitoneum in rats. *Oncotarget* 9(2)**,** 2631-2645. doi: 10.18632/oncotarget.23498.

Fukuda, K., Asoh, S., Ishikawa, M., Yamamoto, Y., Ohsawa, I., and Ohta, S. (2007). Inhalation of hydrogen gas suppresses hepatic injury caused by ischemia/reperfusion through reducing oxidative stress. *Biochem Biophys Res Commun* 361(3)**,** 670-674. doi: 10.1016/j.bbrc.2007.07.088.

Gao, Y., Yang, H., Fan, Y., Li, L., Fang, J., and Yang, W. (2016). Hydrogen-Rich Saline Attenuates Cardiac and Hepatic Injury in Doxorubicin Rat Model by Inhibiting Inflammation and Apoptosis. *Mediators Inflamm* 2016**,** 1320365. doi: 10.1155/2016/1320365.

Ge, Y.S., Zhang, Q.Z., Li, H., Bai, G., Jiao, Z.H., and Wang, H.B. (2019). Hydrogen-rich saline protects against hepatic injury induced by ischemia-reperfusion and laparoscopic hepatectomy in swine. *Hepatobiliary Pancreat Dis Int* 18(1)**,** 48-61. doi: 10.1016/j.hbpd.2018.12.001.

Iketani, M., Ohshiro, J., Urushibara, T., Takahashi, M., Arai, T., Kawaguchi, H., et al. (2017). Preadministration of Hydrogen-Rich Water Protects Against Lipopolysaccharide-Induced Sepsis and Attenuates Liver Injury. *Shock* 48(1)**,** 85-93. doi: 10.1097/SHK.0000000000000810.

Ishikawa, T., Shimada, S., Fukai, M., Kimura, T., Umemoto, K., Shibata, K., et al. (2018). Post-reperfusion hydrogen gas treatment ameliorates ischemia reperfusion injury in rat livers from donors after cardiac death: a preliminary study. *Surg Today* 48(12)**,** 1081-1088. doi: 10.1007/s00595-018-1693-0.

Koyama, Y., Taura, K., Hatano, E., Tanabe, K., Yamamoto, G., Nakamura, K., et al. (2014). Effects of oral intake of hydrogen water on liver fibrogenesis in mice. *Hepatol Res* 44(6)**,** 663-677. doi: 10.1111/hepr.12165.

Li, H., Bai, G., Ge, Y., Zhang, Q., Kong, X., Meng, W., et al. (2018a). Hydrogen-rich saline protects against small-scale liver ischemia-reperfusion injury by inhibiting endoplasmic reticulum stress. *Life Sci* 194**,** 7-14. doi: 10.1016/j.lfs.2017.12.022.

Li, S., Fujino, M., Ichimaru, N., Kurokawa, R., Hirano, S., Mou, L., et al. (2018b). Molecular hydrogen protects against ischemia-reperfusion injury in a mouse fatty liver model via regulating HO-1 and Sirt1 expression. *Sci Rep* 8(1)**,** 14019. doi: 10.1038/s41598-018-32411-4.

Liu, Q., Li, B.S., Song, Y.J., Hu, M.G., Lu, J.Y., Gao, A., et al. (2016). Hydrogen-rich saline protects against mitochondrial dysfunction and apoptosis in mice with obstructive jaundice. *Mol Med Rep* 13(4)**,** 3588-3596. doi: 10.3892/mmr.2016.4954.

Liu, Q., Shen, W.F., Sun, H.Y., Fan, D.F., Nakao, A., Cai, J.M., et al. (2010). Hydrogen-rich saline protects against liver injury in rats with obstructive jaundice. *Liver Int* 30(7)**,** 958-968. doi: 10.1111/j.1478-3231.2010.02254.x.

Lu, Z., Lin, Y., Peng, B., Bao, Z., Niu, K., and Gong, J. (2017). Hydrogen-Rich Saline Ameliorates Hepatic Ischemia-Reperfusion Injury Through Regulation of Endoplasmic Reticulum Stress and Apoptosis. *Dig Dis Sci* 62(12)**,** 3479-3486. doi: 10.1007/s10620-017-4811-8.

Matsuno, N., Watanabe, R., Kimura, M., Iwata, S., Fujiyama, M., Kono, S., et al. (2014). Beneficial effects of hydrogen gas on porcine liver reperfusion injury with use of total vascular exclusion and active venous bypass. *Transplant Proc* 46(4)**,** 1104-1106. doi: 10.1016/j.transproceed.2013.11.134.

Shimada, S., Wakayama, K., Fukai, M., Shimamura, T., Ishikawa, T., Fukumori, D., et al. (2016). Hydrogen Gas Ameliorates Hepatic Reperfusion Injury After Prolonged Cold Preservation in Isolated Perfused Rat Liver. *Artif Organs* 40(12)**,** 1128-1136. doi: 10.1111/aor.12710.

Sun, H., Chen, L., Zhou, W., Hu, L., Li, L., Tu, Q., et al. (2011). The protective role of hydrogen-rich saline in experimental liver injury in mice. *J Hepatol* 54(3)**,** 471-480. doi: 10.1016/j.jhep.2010.08.011.

Tan, Y.C., Xie, F., Zhang, H.L., Zhu, Y.L., Chen, K., Tan, H.M., et al. (2014). Hydrogen-rich saline attenuates postoperative liver failure after major hepatectomy in rats. *Clin Res Hepatol Gastroenterol* 38(3)**,** 337-345. doi: 10.1016/j.clinre.2013.11.007.

Uto, K., Sakamoto, S., Que, W., Shimata, K., Hashimoto, S., Sakisaka, M., et al. (2019). Hydrogen-rich solution attenuates cold ischemia-reperfusion injury in rat liver transplantation. *BMC Gastroenterol* 19(1)**,** 25. doi: 10.1186/s12876-019-0939-7.

Xiang, L., Tan, J.W., Huang, L.J., Jia, L., Liu, Y.Q., Zhao, Y.Q., et al. (2012). Inhalation of hydrogen gas reduces liver injury during major hepatotectomy in swine. *World J Gastroenterol* 18(37)**,** 5197-5204. doi: 10.3748/wjg.v18.i37.5197.

Yan, M., Yu, Y., Mao, X., Feng, J., Wang, Y., Chen, H., et al. (2019). Hydrogen gas inhalation attenuates sepsis-induced liver injury in a FUNDC1-dependent manner. *Int Immunopharmacol* 71**,** 61-67. doi: 10.1016/j.intimp.2019.03.021.

Yang, S.C., Chen, L.L., Fu, T., Li, W.Y., and Ji, E.S. (2018). [Improvement of hydrogen on liver oxidative stress injury in chronic intermittent hypoxia rats]. *Zhongguo Ying Yong Sheng Li Xue Za Zhi* 34(1)**,** 61-64. doi: 10.12047/j.cjap.5484.2018.016.

Zhang, C.B., Tang, Y.C., Xu, X.J., Guo, S.X., and Wang, H.Z. (2015a). Hydrogen gas inhalation protects against liver ischemia/reperfusion injury by activating the NF-kappaB signaling pathway. *Exp Ther Med* 9(6)**,** 2114-2120. doi: 10.3892/etm.2015.2385.

Zhang, J.Y., Song, S.D., Pang, Q., Zhang, R.Y., Wan, Y., Yuan, D.W., et al. (2015b). Hydrogen-rich water protects against acetaminophen-induced hepatotoxicity in mice. *World J Gastroenterol* 21(14)**,** 4195-4209. doi: 10.3748/wjg.v21.i14.4195.

Zhang, Q., Ge, Y., Li, H., Bai, G., Jiao, Z., Kong, X., et al. (2018). Effect of hydrogen-rich saline on apoptosis induced by hepatic ischemia reperfusion upon laparoscopic hepatectomy in miniature pigs. *Res Vet Sci* 119**,** 285-291. doi: 10.1016/j.rvsc.2018.07.005.

Zhang, Q., Piao, C., Xu, J., Jiao, Z., Ge, Y., Liu, X., et al. (2019). Comparative study on protective effect of hydrogen rich saline and adipose-derived stem cells on hepatic ischemia-reperfusion and hepatectomy injury in swine. *Biomed Pharmacother* 120**,** 109453. doi: 10.1016/j.biopha.2019.109453.
